# Ultrafast thermal-free photoluminescence of coherently extended single quantum states

**DOI:** 10.1038/s41598-019-44940-7

**Published:** 2019-06-11

**Authors:** Takuya Matsuda, Masayoshi Ichimiya, Masaaki Ashida, Hajime Ishihara

**Affiliations:** 1 Department of Physics and Electronics, Osaka Prefecture University, 1-1 Gakuencho, Naka-ku, Sakai, Osaka, 599-8531 Japan; 2 Institute for Solid State Physics, University of Tokyo, Kashiwa, Chiba, 277-8581 Japan; 3 Department of Electronic Systems Engineering, The University of Shiga Prefecture, Hikone, Shiga, 522-8533 Japan; 4 Department of Materials Engineering Science, Osaka University, Toyonaka, Osaka, 560-8531 Japan

**Keywords:** Polaritons, Optical spectroscopy

## Abstract

The coherent volume of single quantum states of matter is typically smaller than that of photons by several orders of magnitude, and hence, interactions between photons and single quantum states are normally very weak. This limits the speed of radiative decay of matter states in free space. Recent efforts to speed-up radiative processes have been focused on creating a small mode volume of photons using cavity systems, or on realizing spontaneous synchronization among quantum emitters to create a dipole at the macroscopic scale, which accelerates photon emission up to a couple of hundred femtoseconds. Here, we demonstrate the 10-fs class of photoluminescence (PL) of a single quantum state in solid thin films without the use of a photo-cavity system or the spontaneous synchronization effect. Significantly, this speed can beat thermal dephasing of relevant excited states at room temperature, which is typically a couple of tens of femtoseconds. The process occurs due to the giant interaction volume between light waves and the multipole excitonic waves. This result indicates the possibility to realize photoemission processes that complete before the thermal dephasing process activates, which opens up the hidden potential of ubiquitous solids as thermal-free or extremely low-energy-loss photonic materials.

## Introduction

Luminescence is one of the most fundamental manifestations of light–matter interaction, and its processes have been significant subjects in a wide variety of photonics and material science fields. Understanding the luminescence processes is crucial for developing efficient and highly functional light sources^1–5^ and for exploiting probes that provide information on the dynamics of various matter excitation states^6^. One active research direction is the pursuit of fast and highly efficient luminescence using the design of material systems. By creating a small mode volume of photons using cavity systems or localized surface plasmons, strong couplings between the emitters and localized modes can be realized, resulting in the Purcell effect that speeds up photon emission^7^. Spontaneous synchronization among radiatively interacting polarizations of quantum emitters has also been realized, where the dipole was formed at the macroscopic scale by the synchronization, and exhibited a burst photoemission (superfluorescence) through giant coupling with photon modes in free space^8–11^. In addition to these strategies, monolayer transition-metal dichalcogenides have also attracted attention recently because of the giant oscillator strength of intrinsic excitonic states, which has a radiative decay time in the 100-fs class^12,13^.

Here, we demonstrate significantly faster luminescence than previously recorded, reaching 10 fs, from a simple sub-micron thin film. We should note that this speed can overcome thermal dephasing of relevant excited states at room temperature, which is typically a couple of tens of femtoseconds^14^. The origin of the fast PL is an ultra-long-range coherent interaction between the multipole excitonic waves and light waves. Experimentally observing the 10-fs class of fast luminescence has been challenging because the extraordinarily wide spectrum of the corresponding state^15^ is completely hidden behind the signals of many other states with much narrower widths. In the present observation, the optical response signal of such ultrafast states alone survives at room temperature, beating the thermal dephasing in the employed material. Therefore, we could successfully identify the 10-fs class of luminescence of a single quantum state in solids by examining the temperature dependence of the PL spectra, which excellently agreed with the theoretical prediction.

In the present study, we used wide-gap semiconductor CuCl, where the excitons, i.e., the bound electron-hole (e–h) pairs, are excited near the ultraviolet region. The excitonic luminescence in this frequency region can be used as white light sources with the help of phosphors as LEDs based on InGaN materials. An extremely fast luminescence shows the possibility to greatly enhance the device efficiency. Further, if the luminescence is faster than the excitonic dephasing at room temperature, this process greatly reduces a heat generation. Thus, in such a case, the compatibility of high efficiency of luminescence and low consumption of energy is possible.

**Figure 1:**
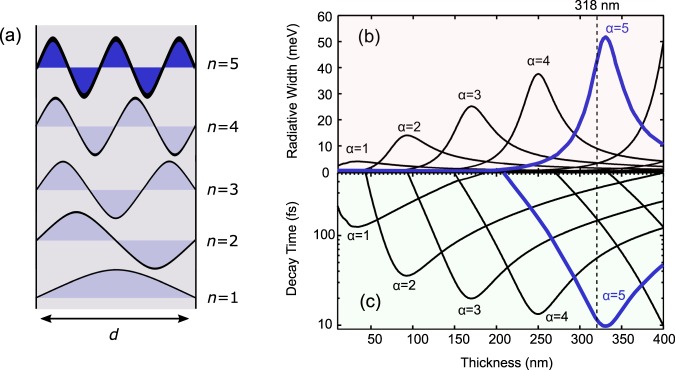
(**a**) Schematic illustration of excitonic wavefunctions confined in a thin film. (**b**) Calculated radiative widths and (**c**) decay time as a function of film thickness in a CuCl thin film.

The effective Bohr radius of the e–h pair of CuCl excitons is several angstroms, and hence, the center-of-mass (c.m.) motion of excitons acts as a principal degree of freedom for the sample volume above the nanometer scale. For the theoretical model, we considered sinusoidal functions of the c.m. motion of the excitons confined in the thickness (*z*) direction, as illustrated in Fig. 1(a). Namely, the kinetic energy *E*_*n*_ and its wavefunction Ψ_*n*_(***r***) can be written as ${{\rm{\Psi }}}_{n}({\boldsymbol{r}})={\varphi }_{1s}{\psi }_{n}({\boldsymbol{r}})={\varphi }_{1s}{S}^{-1/2}{e}^{{\rm{i}}{{\boldsymbol{K}}}_{\parallel }\cdot {{\boldsymbol{r}}}_{\parallel }}{(2/d)}^{1/2}\,\sin ({K}_{n}z)$ and ${E}_{n}={\hbar }{\omega }_{{\rm{T}}}+{{\hbar }}^{2}({K}_{n}^{2}+{K}_{\parallel }^{2})/2M$, where *ϕ*_1s_ is the e-h relative wavefunction of the 1 s state, *S* is an area along the film surface, ***K***_∥_ and ***r***_∥_ are the wavevector and position vector parallel to the film surface, respectively, *d* is the film thickness, *K*_*n*_ is the quantized wavenumber perpendicular to the film surface whose quantization condition is given as *K*_*n*_ = *nπ*/*d* with positive integer *n*, *ℏω*_T_ is the energy of the bulk transverse exciton, and *M* is the total mass of an exciton. Each quantum state *λ* is labelled by *n*, ***K***_∥_, and polarization direction *ξ*(=*x*, *y*, *z*) as *λ* = |*n*, ***K***_∥_, *ξ*〉. Hereafter, we consider the subspace of ***K***_∥_ = 0 and fix the polarization direction. The induced polarization can be expanded by the basis of the above exciton states as $P(z,\omega )=\sum _{n}\,{X}_{n}(\omega ){\rho }_{0n}(z)$, where the matrix element of the transition dipole density ***ρ***_0*n*_(*z*) of this model is ***ρ***_0*n*_(*z*) = *μ*(2/*d*)^1/2^sin(*K*_*n*_*z*). In this expression, *μ* is given by *μ*^2^ = *ε*_bg_Δ_LT_/4*π*, where *ε*_bg_ is the background dielectric constant of the medium and Δ_LT_ is the split energy between the transverse excitonic energy *ℏω*_T_ and the longitudinal excitonic energy *ℏω*_L_ in the bulk. By substituting *P*(*z*, *ω*) into the microscopic Maxwell equation, we can obtain a set of linear equations to determine {*X*_*n*_(*ω*)}. This set of equations can be written in matrix form with the coefficient matrix *S*(*ω*). The details of the calculations are provided in the Methods section. The roots of det{*S*(*ω*)} = 0 provide the complex eigenenergies {Ω_*α*_} of the coupled radiation–matter system. These modes not only include self-energy (radiative) correction but also diagonalize the coupling between different exciton states via radiation. Thus, these modes form new eigenmodes {*α*} whose real parts {*Re*[Ω_*α*_]} include the radiative energy shift and whose imaginary parts {*Im*[Ω_*α*_]} correspond to the radiative width.

## Results

Figure 1(b,c) show the calculated radiative width and decay time of the light–exciton coupled system ({*Im*[Ω_*α*_]}) with the material parameters for CuCl (see the Methods section). We can see the resonant size enhancement of the radiative width for each eigenmode. In the conventional understanding of the excitonic optical response, only the dipole type state couples with light because the light wavelength is much longer than the spatial expansion of the excitonic wavefunctions. In contrast with such a picture of the optical response of solids, multipole-type excitons (*n* > 1) coherently extend over the entire submicron-scale sample if the sample quality is sufficiently high (see, Fig. 1(a)), where the interplay between the excitonic waves (of non-dipole type states) and the light waves becomes important. In the present sample parameters, approximately one wavelength of light comes inside the film at around 100 nm thickness of the film because of the large background dielectric function. Thus, *n* = 2 state (in Fig. 1(a)) strongly couples with light and the radiative width becomes maximum at this thickness (see, Fig. 1(b)). If the thickness becomes further larger, a greater number of waves of light come inside the film, and then, the coupling between the light and *n* = 3 state becomes strong instead of *n* = 2 state, and its radiative width becomes larger. For the sample thickness used in the present study (318 nm), the radiative width of *n* = 5 becomes largest. In this way, multipole-type excitons (*n* > 1) can coherently couple with light fields, leading to the coupled modes with giant radiative corrections (shift and width), as seen in Fig. 1(b,c). The existence of such modes has been proposed theoretically^16–22^, where some coupled modes have a very large imaginary part corresponding to the 100-fs class of ultrafast radiative decay. In fact, the 100-fs class of ultrafast radiative decay has been observed using a nonlinear optical technique^23^. Another recent study has more systematically revealed the existence of long-range light–exciton coupling in thin films^15^ by observing the real part including the radiative shift. However, in spite of these recent experimental successes, demonstrating the 10-fs class of luminescence, whose existence is predicted as in Fig. 1 (see the blue wave in (a) and blue lines in (b, c)), still seems to be challenging because of its extremely wide width in the spectrum, as discussed in ref.^15^.

In the present study, we utilize a particular nature of excitons in CuCl that hardly show PL at room temperature because fast dephasing due to strong exciton–phonon coupling prohibits their luminescence. We should note that if there is a mode radiating faster than the dephasing, the luminescence of such a mode will appear without being hidden by other sharp modes. In order to realize this situation, we use a CuCl film with a thickness of 318 nm. (see the mode of *α* = 5 in Fig. 1(b,c)). Measurement methods are described in the Methods section.

**Figure 2:**
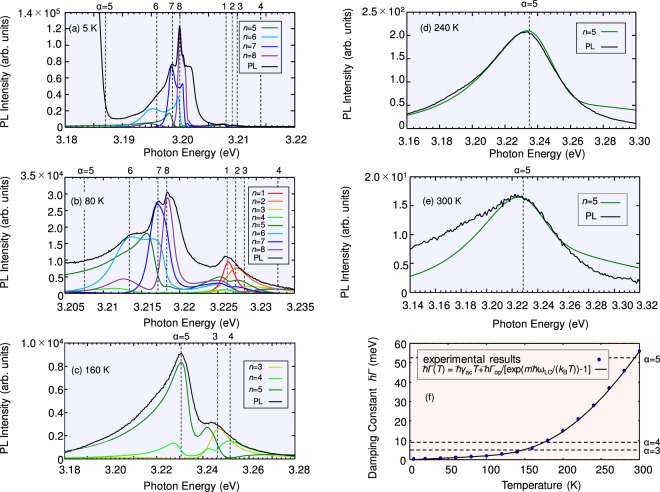
Measured PL spectra (black solid lines) at (**a**) 5 K, (**b**) 80 K, (**c**) 160 K, (**d**) 240 K, and (**e**) 300 K for a CuCl thin film with a thickness of 318 nm. Coloured solid lines show the spectra of the induced polarization associated with respective *n*th confined excitons. (**f**) Temperature dependence of the damping constant *ℏ*Γ. Blue dots represent the values determined by the fitting, and the black solid line is derived from the equation *ℏ*Γ(*T*) = *ℏγ*_ac_*T* + *ℏ*Γ_op_/[exp[*mℏΩ*_LO_/(*k*_B_*T*)] − 1](*m* ∈ ${\mathbb{N}}$).

In Fig. 2(a–e), we show the measured PL spectra (black solid line) and plot the calculated induced-polarization spectra associated with *n*th original excitonic state (coloured solid line) at each temperature. As shown in Fig. 2(a), the shape of the PL spectrum closely reflects the spectra of the induced-polarization. (The band-gap energy shift depending on temperature was safely estimated in the fitting of the reflection spectra).

The induced polarization of each *n*th excitonic state includes components of the diagonalized light–exciton coupled modes {*α*}, and peaks corresponding to these modes appear in each spectrum, where the dominant contribution is given from the same quantum number, i.e., *n* = *α*. The well-fitted spectral peaks indicate that PL occurs at coherently coupled light–exciton modes^24^. From Fig. 2(a–d), we observe the following behaviours. At low temperature, structures with relatively sharp peaks are dominant, and wider structures are difficult to see. At 5 K, in *n* = 5 polarization, the sharp component from *α* = 8 mode can be seen though the main component, i.e., *α* = 5 mode with very wide width (see Table 1) cannot be recognized. On the other hand, at 80 K and 160 K, the intensities of the sharp-peak structures are suppressed by nonradiative damping, and structures for wide radiative width become relatively apparent. Competition between the radiative width of each mode and nonradiative damping, whose derivation is explained later, at each temperature can be seen in Fig. 2(f). Only *α* = 5 mode can beat nonradiative damping above 240 K in Fig. 2(f). Actually, in Fig. 2(d,e), only the peak structure from *α* = 5 mode appears, which can be well-fitted by the fifth induced polarization predominantly containing *α* = 5 mode at this temperature region.

**Table 1:** Calculated eigenenergies at 0 K and radiative width of respective light–exciton coupled modes in a CuCl thin film with a thickness of 318 nm.

Eigenstate *α*	1	2	3	4	5	6	7	8
Eigenenergy [eV] at 0 K	3.2082	3.209	3.2105	3.2143	**3.1875**	3.1962	3.199	3.2002
Radiative width [meV]	1.0	2.8	4.4	9.4	52	3.4	1.0	0.57

## Discussion

In the above fitting, we treated nonradiative damping *ℏ*Γ phenomenologically as a fitting parameter. The fitted values are plotted in Fig. 2(f) in blue dots. On the other hand, *ℏ*Γ can be expressed as *ℏ*Γ(*T*) = *ℏγ*_ac_*T* + *ℏ*Γ_op_/[exp[*mℏΩ*_LO_/(*k*_B_*T*)] − 1](*m* ∈ ${\mathbb{N}}$)^25^, where Ω_LO_ is the longitudinal optical (LO) phonon frequency, *ℏγ*_ac_ is the coupling strength of the exciton–acoustic phonon interaction, *ℏ*Γ_op_ is a parameter describing the strength of the exciton–LO phonon interaction, *k*_B_ is the Boltzmann constant, and *T* is the temperature. The curve corresponding to this equation is also plotted in Fig. 2(f) with the parameters in ref.^25^: $m=3,{\hbar }{\gamma }_{{\rm{ac}}}=19.5\,\mu \mathrm{eV}/K$, and *ℏ*Γ_op_ = 976 meV. The number *m* = 3 suggests that the exciton–LO phonon interactions involve almost three phonons^26^. The values determined in the fitting are in excellent agreement with this equation, which implies that, for the present CuCl crystals, our derived nonradiative damping can be explained in terms of the phonon modes by comparison to the data in ref.^25^. As shown in Fig. 2(f), the damping constant of excitons is 56 meV, particularly at 300 K, whose value is estimated as 12 fs = *ℏ*/(56 meV). On the other hand, as shown in Table 1, the radiative width of the *α* = 5 mode is 52 meV, whose value is estimated as 13 fs = *ℏ*/(52 meV). In other words, there is a competition between the radiative decay and the dephasing at 300 K. Thus, we have successfully educed ultrafast PL that overcomes thermal dephasing at room temperature.

**Figure 3:**
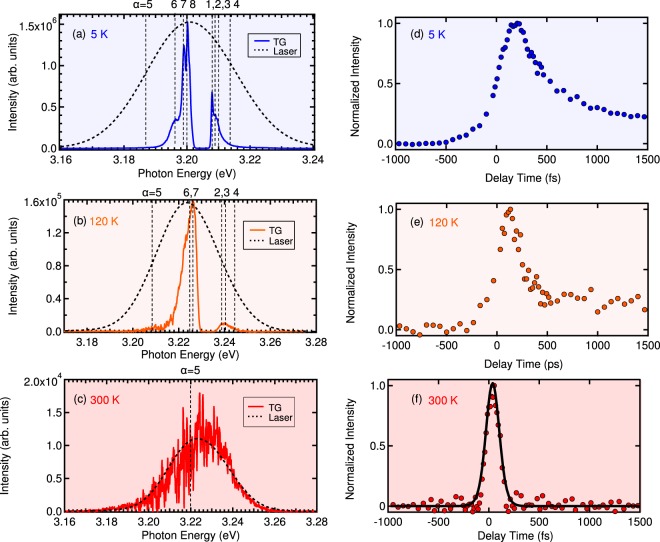
(**a**–**c**) The TG spectra at 5 K, 120 K, and 300 K for the same CuCl thin film as those in the PL measurement. The photon energy range in each figure is set by considering the shift of excitonic energies estimated from the fitting of the reflectance spectra. The vertical dotted lines indicate the eigenenergies of exciton–light coupled modes {*α*}. (**d**–**f**) Delay time dependence of TG signal intensity corresponding to the respective left panels. The dashed line in (**f**) represents the fitting of a single-exponential function convoluted by the excitation laser profile.

**Figure 4:**
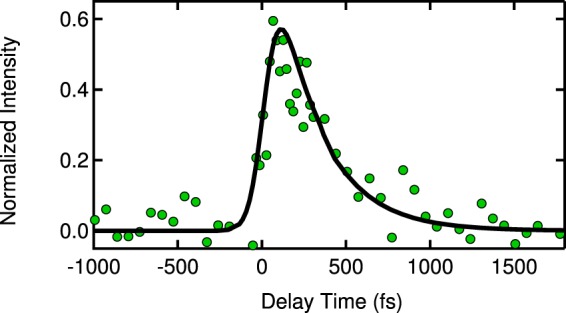
Fitting of the TG time profile. The curve is given by subtracting the value for 120 K from that for 5 K. The fitting is performed by using two decay constants for *α* = 1 and *α* = 8.

Finally, we corroborate the above demonstration using a transient grating (TG) measurement. The TG spectroscopy is a kind of four-wave mixing (FWM) spectroscopies based on the third order nonlinearity, where a variety of enhanced versions of FWM spectroscopies have been developed recently^27,28^. The TG spectroscopy is a standard method to investigate the population damping of excitons^29–32^, where the two short pump pulses with wavevectors ***k***_1_ and ***k***_2_ form a transient grating of the excitation probability (population of excitons) by their interference. A diffracted pulse (with ***k***_3_ + ***k***_1_ − ***k***_2_ or ***k***_3_ + ***k***_2_ − ***k***_1_) by this grating appears from the probe pulse with wavevector ***k***_3_ and perpendicular polarization to that of the first and second pulses, avoiding the coherent signal of the FWM, when the population grating exists. The intensity of the diffracted light depends on the intensity of the grating. Thus, by observing the delay time dependence of the TG signal, we can measure the population decay time. The measurement method and experimental setup are explained in the Methods section. Figure 3(a–f) show, respectively, the TG spectra and the delay time dependence *τ*_23_ (see the explanation of TG measurement in the Methods section for details of *τ*_23_) of TG signal intensities at 5 K, 120 K, and 300 K for the same CuCl thin film as that in PL measurement). The TG spectra also exhibit several peaks corresponding to the exciton–light coupled modes {*α*}. In the TG spectra, with increasing temperature, the slower modes disappear, and the faster modes survive. In particular, at 300 K, the ultrafast mode *α* = 5 survives in the TG spectrum, as shown in Fig. 3(c). In the real-time domain, at 5 K, we find that many slower modes contribute to the TG intensity as slow decay components, as shown in Fig. 3(d). On the other hand, with the increase in temperature, the slower modes disappear, and only the faster modes contribute to the TG time profile. This situation can be seen more clearly in Fig. 4, which shows the curve given by subtracting the TG of 120 K from that of 5 K. The fitting was performed with two exponential functions for decay constants of *α* = 1 and *α* = 8 convoluted by the excitation laser profile. This successful fitting clearly indicates that the change of the decay profile by temperature was due to the change of the *α* modes involved in the coherent TG process. At 300 K in Fig. 3(f), the time profile can be fitted by a single-exponential function. The decay time estimated by this fitting falls into the 10 fs to 20 fs range, which is consistent with the radiative width of the *α* = 5 mode. For details of this fitting, see the Methods section.

## Conclusion

In conclusion, we established, for the first time, the 10-fs class of ultrafast PL that was faster than the thermal dephasing of electronic excited s in solid thin films. In contrast with conventional luminescence pictures, the present PL arises from the coherent light–exciton coupled modes with sub-micron-scale interaction volumes, where multipole-type excitons (*n* > 1) are coupled with the light waves. The present result opens the possibility of thermal-free operation of the 10-fs class of photo-signals using ubiquitous solid materials, which is significant for future ultrafast and low-energy-loss photo-technologies. Although the decay process from the excited carriers to the observed excitons is not clear in the present study, the PL efficiency is expected to be significantly enhanced under the resonant excitation, which is a crucial subject for a future study. Further study of similar effects in materials with larger oscillator strengths of molecular crystals (such as anthracene or tetracene) is desired because the larger radiative shift results in energetic isolation of the thermal-free modes from other slow modes, leading to true thermal-free photonic operations.

## Methods

### Theoretical method

We consider a thin film whose direction of thickness was along the *z*-axis. This film confines the c.m. motion of the excitons. Assuming the Bohr radius to be much smaller than the film thickness, we explicitly treat only the degree of freedom of the c.m. motion, and the relative motion is treated as that in the bulk. In other words, we do not consider distortions of the c.m. wavefunctions near the film surface. The following eigenfunctions and eigenenergies are used as the basis diagonalizing the kinetic energy part of excitons: ${{\rm{\Psi }}}_{n}({\boldsymbol{r}})={\varphi }_{1s}{\psi }_{n}({\boldsymbol{r}})={\varphi }_{1s}{S}^{-1/2}{e}^{{\rm{i}}{{\boldsymbol{K}}}_{\parallel }\cdot {{\boldsymbol{r}}}_{\parallel }}{(2/d)}^{1/2}\,\sin ({K}_{n}z)$, ${E}_{n}={\hbar }{\omega }_{{\rm{T}}}+{{\hbar }}^{2}({K}_{n}^{2}+{K}_{\parallel }^{2})/2M$, where *ϕ*_1s_ is the relative wavefunction of the 1 s state, *S* is an area along the film surface, ***K***_∥_ and ***r***_∥_ are the wavevector and position vector parallel to the film surface, respectively, *d* is the film thickness, *K*_*n*_ is the quantized wavenumber perpendicular to the film surface whose quantization condition is given as *K*_*n*_ = *nπ*/*d* with a positive integer *n*, *ℏω*_T_ is the energy of the bulk transverse exciton, and *M* is the total mass of an exciton. Each quantum state was labelled by *n*, *K*_∥_, and polarization. From these excitonic states, we can calculate the linear nonlocal susceptibility in terms of the matrix element of the transition dipole density ***ρ***_0*n*_(***r***) as, $\bar{\chi }({\boldsymbol{r}},{\boldsymbol{r}}^{\prime} ;\omega )=\sum _{n}\,{\rho }_{0n}({\boldsymbol{r}}){\rho }_{n0}({\boldsymbol{r}}^{\prime} )/({E}_{n}-{\hbar }\omega -{\rm{i}}{\hbar }{\rm{\Gamma }})$, being based on the linear response theory, and the constitutive equation can be obtained as $P({\boldsymbol{r}},\omega )=\int {\rm{d}}{\boldsymbol{r}}^{\prime} \bar{\chi }({\boldsymbol{r}},{\boldsymbol{r}}^{\prime} ;\omega ){\boldsymbol{E}}({\boldsymbol{r}},\omega )$, where *P*(***r***, *ω*) and ***E***(***r***, *ω*) are the induced polarization and the self-consistent electric field, respectively. Solving the Maxwell equation including the above induced polarization, we obtain the expression of the self-consistent field as^33,34^

1 $${\boldsymbol{E}}({\boldsymbol{r}},\omega )={{\boldsymbol{E}}}_{0}({\boldsymbol{r}},\omega )+\sum _{n}\,\int {\rm{d}}{\boldsymbol{r}}^{\prime} \int {\rm{d}}{\boldsymbol{r}}^{\prime\prime} G({\boldsymbol{r}},{\boldsymbol{r}}^{\prime} ;\omega )\frac{{{\boldsymbol{\rho }}}_{0n}({\boldsymbol{r}}^{\prime} ){\rho }_{n0}({\boldsymbol{r}}^{\prime\prime} )}{{E}_{n}-\hslash \omega -{\rm{i}}\hslash {\rm{\Gamma }}}{\boldsymbol{E}}({\boldsymbol{r}}^{\prime\prime} ,\omega ),$$

where ***E***_0_(***r***, *ω*) is the incident field, *G*(***r***, ***r***′; *ω*) is the dyadic Green’s function satisfying the equation.

[∇ × ∇ × − *q*^2^*ε*_bg_(***r***)]*G*(***r***, ***r***′; *ω*) = 4*πq*^2^$\bar{{\bf{I}}}$*δ*(***r*** − ***r***′), ***ρ***_*n*0_(***r***) is the matrix element of the transition dipole between the ground state and *n*-th exciton, and *ℏ*Γ is a phenomenologically introduced nonradiative damping constant of excitons. In this equation, $\bar{{\bf{I}}}$ is the unit dyad, and *ε*_bg_(***r***) is the background dielectric constant, which includes information about the film geometry. We used the Green’s function that reflected the spatial structure of this dielectric constant^35^. Defining the quantity ${X}_{n}(\omega )\equiv {({E}_{n}-{\hbar }\omega -{\rm{i}}{\hbar }{\rm{\Gamma }})}^{-1}\int {\rm{d}}{\boldsymbol{r}}{{\boldsymbol{\rho }}}_{n0}({\boldsymbol{r}})\cdot {\boldsymbol{E}}({\boldsymbol{r}},\omega )$, we could rewrite the above Maxwell equation into a linear equation system to determine *X*_*n*_(*ω*) as

2 $$({E}_{n}-\hbar \omega -{\rm{i}}\hbar {\rm{\Gamma }}){X}_{n}(\omega )+\sum _{n^{\prime} }\,{X}_{n^{\prime} }(\omega ){A}_{n,n^{\prime} }(\omega )={X}_{n}^{(0)}(\omega ),$$

where ${X}_{n}^{(0)}(\omega )=\int {\rm{d}}{\boldsymbol{r}}{{\boldsymbol{\rho }}}_{n0}({\boldsymbol{r}})\cdot {{\boldsymbol{E}}}_{0}({\boldsymbol{r}})$, and ${A}_{n,n^{\prime} }(\omega )=-\,\int {\rm{d}}{\boldsymbol{r}}\int {\rm{d}}{\boldsymbol{r}}^{\prime} {{\boldsymbol{\rho }}}_{n0}({\boldsymbol{r}})\cdot {\bf{G}}({\boldsymbol{r}},{\boldsymbol{r}}^{\prime} ;\omega )\cdot {{\boldsymbol{\rho }}}_{0n^{\prime} }({\boldsymbol{r}}^{\prime} )$. ${A}_{n,n^{\prime} }(\omega )$ means the interaction between the induced polarizations originating from the *n*th and $n^{\prime}$th states. From the definition of ${\bf{G}}({\boldsymbol{r}},{\boldsymbol{r}}^{\prime} ;\omega )$, ${A}_{n,n^{\prime} }(\omega )$ includes both the retarded and instantaneous Coulomb interactions. Expressing Eq. (2) in matrix form as $S(\omega ){\bf{X}}(\omega )={{\bf{X}}}^{(0)}({\boldsymbol{\omega }})$, where *S*(*ω*) is the coefficient matrix, the roots of det *S*(*ω*) = 0 provide the complex eigenmodes {Ω_*α*_} of the coupled system whose real parts {Re[Ω_*α*_]} include the radiative energy shift and whose imaginary parts {Im[Ω_*α*_]} correspond to the radiative decay rate.

### Parameters used in calculations

Here, *M* = 2.3*m*_0_, where *m*_0_ is the electron mass, *ℏω*_T_ = 3.202 eV, *ε*_bg_ = 5.59, and Δ_LT_ = 5.7 meV.

### PL measurement

A CuCl film with a thickness of 318 nm was grown by molecular-beam epitaxy. The film was mounted in a helium flow cryostat, and the temperature was set from 5 K to 300 K. We employed a diode-pumped solid-state laser with a wavelength of 320 nm (~3.87 eV) and a power of 10 mW. This photon energy is much higher than the *Z*_3_ transverse exciton energy (3.202 eV) of bulk CuCl at 5 K. The PL spectra were detected in a backward scattering configuration, and the signal was transmitted through an optical fibre to a monochromator equipped with a nitrogen-cooled charge-coupled device (CCD). The spectral resolution was better than 0.1 meV.

### TG measurement

**Figure 5:**
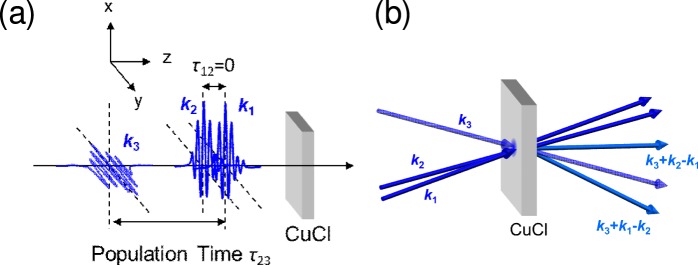
(**a**) Time-ordering and (**b**) schematic in the transient grating (TG) experiment. Here, ***k***_1_ and ***k***_2_ are *x*-polarized light, and ***k***_3_ is *y*-polarized light. Population time *τ*_23_ corresponds to the delay time between ***k***_1_, ***k***_2_, and ***k***_3_. TG signals correspond to ***k***_3_ + ***k***_2_ − ***k***_1_ and ***k***_3_ + ***k***_1_ − ***k***_2_.

The TG signals were measured using the second harmonic of a mode-locked Ti:sapphire laser, which had a repetition rate and pulse duration of 80 MHz and 60 fs, respectively. The spectral width of the excitation light was so wide to reach ~4 meV that the whole energy region of the exciton resonance was covered. The light was split into three pulses, and all of them were focused onto the same spot on the sample surface. In the TG configuration, polarization of the probe pulse was perpendicular to that of the parallel-polarized pump pulses as illustrated in Fig. 5(a). The signal light was transmitted through an optical fibre to the monochromator equipped with the CCD. As depicted in Fig. 5(b), the three pulses were incident on the sample in a specific time order in which pulses ***k***_1_ and ***k***_2_ simultaneously arrived at the sample followed by pulse ***k***_3_. Delays between pump pulses ***k***_1_ and ***k***_2_ and probe pulse ***k***_3_ in the emitted TG signal were denoted as *τ*_23_ (Fig. 5(a)).

**Figure 6:**
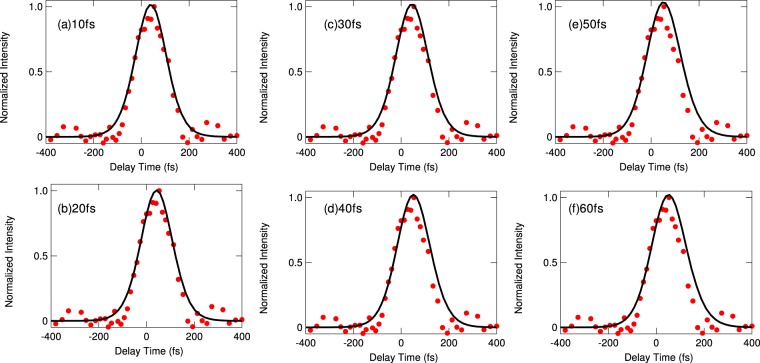
Parameter dependence of the fitting with the observed delay-time dependence of the TG signal at 300 K, which is the same as that in Fig. 3(f). The decay times from 10 fs to 60 fs are examined.

In the fitting of the TG delay time profile, we first estimated the excitation pulse duration to be 120 fs using the cross-correlation between the fundamental and second harmonics of the laser. Then, considering that the TG process is a third-order optical response, three-fold integration was performed in the convolution calculation between the excitation pulse profile and the assumed TG delay-time profile with a single-exponential function. Because the excitation pulse duration was longer than the decay time, we performed the fitting very carefully, as shown in Fig. 6. In this figure, we can see that the decay time falls into the 10 fs–20 fs range^13^.
